# Effectiveness of a Patient-Tailored, Pharmacist-Led Intervention Program to Enhance Adherence to Antihypertensive Medication: The CATI Study

**DOI:** 10.3389/fphar.2018.01057

**Published:** 2018-09-26

**Authors:** Danielle M. van der Laan, Petra J. M. Elders, Christel C. L. M. Boons, Giel Nijpels, Liset van Dijk, Jacqueline G. Hugtenburg

**Affiliations:** ^1^Department of Clinical Pharmacology and Pharmacy and the Amsterdam Public Health Research Institute, VU University Medical Center, Amsterdam, Netherlands; ^2^Department of General Practice and Elderly Care Medicine and the Amsterdam Public Health Research Institute, VU University Medical Center, Amsterdam, Netherlands; ^3^Netherlands Institute for Health Services Research, Utrecht, Netherlands

**Keywords:** medication adherence, patient-tailored, community pharmacies, randomized controlled trial, antihypertensive medication

## Abstract

**Introduction:** Non-adherence to medication is a complex health care problem. In spite of substantial efforts, up till now little progress has been made to effectively tackle the problem with adherence-enhancing interventions. The aim of this study was to investigate the effectiveness of a patient-tailored, pharmacist-led and theory-driven intervention program aimed to enhance self-reported adherence to antihypertensive medication.

**Materials and Methods:** A parallel-group randomized controlled trial in 20 community pharmacies with nine months follow-up was conducted. Patients (45–75 years) using antihypertensive medication and considered non-adherent based on both pharmacy dispensing data and a self-report questionnaire were eligible to participate. The intervention program consisted of two consultations with the pharmacist to identify participants’ barriers to adhere to medication and to counsel participants in overcoming these barriers. The primary outcome was self-reported medication adherence. Secondary outcomes were beliefs about medicines, illness perceptions, quality of life and blood pressure. Mixed-model and generalized estimating equation (GEE) analyses were used to assess overall effects of the intervention program and effects per time point.

**Results:** 170 patients were included. No significant differences between intervention and control groups were found in self-reported adherence, quality of life, illness perceptions, beliefs about medicines (concern scale), and blood pressure. After nine months, intervention participants had significantly stronger beliefs about the necessity of using their medicines as compared to control participants (mean difference 1.25 [95% CI: 0.27 to 2.24], *p* = 0.012).

**Discussion:** We do not recommend to implement the intervention program in the current form for this study population. Future studies should focus on how to select eligible patient groups with appropriate measures in order to effectively target adherence-enhancing interventions.

**Trial Register:** NTR5017 http://www.trialregister.nl/trialreg/admin/rctview.asp?TC=5017.

## Introduction

Non-adherence to medication is an ever continuing complex and multidimensional health care problem. Adherence is defined as the process by which patients take their medication as agreed upon with their health care provider, composed of initiation, implementation and discontinuation (Vrijens et al., 2012). At these three phases non-adherent behavior could occur. Patients may not take the first dose of a prescribed medicine, deviate from the prescribed dosing regimen for example by taking less or skipping pills or discontinue treatment prematurely. Research shows that adherence to antihypertensive medication is suboptimal (Kronish et al., 2011; Naderi et al., 2012; Abegaz et al., 2017). This results in poorly controlled hypertension, higher risk of cardiovascular events, and increased health care costs (Dragomir et al., 2010; Chowdhury et al., 2013; Kim et al., 2016). In Netherlands, community pharmacists are in the ideal position to address adherence, since they are medicines experts in primary care and the majority of patients receive their medication from a community pharmacy. In the past decades, numerous interventions in community pharmacies have been developed in order to increase medication adherence and improve treatment results (Altowaijri et al., 2013; Cheema et al., 2014). Unfortunately, in most studies the results on the effectiveness of these (pharmacist-led) interventions were disappointing (McDonald et al., 2002; Kripalani et al., 2007; van Dulmen et al., 2007; Linn et al., 2011; Vervloet et al., 2012; Nieuwlaat et al., 2014). In only half of the studies the medication adherence was increased and in only a few studies the intervention led to improved treatment outcomes. One likely explanation is that most studies did not use a theoretical framework, crucial for understanding the complexities of adherence behavior. On top of that, most described interventions did not made an effort to apply a patient-tailored approach for identifying the specific causes or barriers for individual patients. Thus, in spite of substantial efforts, up till now little progress has been made to effectively tackle the persistent problem of medication non-adherence.

Because non-adherence is caused by multiple factors, categorized by the World Health Organization (WHO) as social/economic, patient-, condition-, therapy-, health care team, and system-related factors (Sabate, 2003), interventions should be patient-tailored and designed according to a theoretical framework. A proven effective framework for behavioral change is the Common Sense Model of Self-regulation (Leventhal et al., 1984, 1992, 1997; Diefenbach and Leventhal, 1996). According to this theory it is thought that patients seek to understand their illness by developing a representation of illness and treatment, which guides their health behavior. For example, if a patient regards hypertension as a problem, the patient will adopt health-related behavior (such as taking antihypertensive medication) in order to cope with the problem. Thus, adherence behavior will be influenced by whether it makes sense given patients’ illness and treatment representations (Meyer et al., 1985; Leventhal et al., 1992; Horne and Weinman, 1998, 2002; Reynolds, 2003; Rajpura and Nayak, 2014). Moreover, given the asymptomatic nature of hypertension and the need for long-term medicine use, patients’ understanding of the illness may be important in achieving long-term medication adherence (Meyer et al., 1985).

Cardiovascular medication non-Adherence Tailored Intervention is a patient-tailored and pharmacist-led intervention program aimed to enhance self-reported adherence to antihypertensive medication, based on the Common Sense Model of Self-regulation (Van der Laan et al., 2017). The CATI intervention program includes informing participants about hypertension and its consequences in order to change illness and treatment representations, identifying participants’ barriers to adhere to medication and providing recommendations and deciding on interventions to overcome the identified barriers. In this paper, we report on the effectiveness of the CATI intervention program to enhance self-reported medication adherence compared to usual care.

## Materials and Methods

### Study Design and Setting

We performed a pragmatic parallel-group randomized controlled trial in 20 community pharmacies in rural and urban regions in Netherlands. Since this trial was pragmatic including more pharmacies was not possible. We included patients between March and June 2016 and followed them for nine months. Patients were randomly assigned to the intervention group executed by the pharmacist or control group executed by the pharmacy technician (allocation ratio 1:1). Blinding to treatment allocation was not possible due to the nature of the intervention. The study protocol has been described in more detail elsewhere (Van der Laan et al., 2017). The Medical Ethics Committee of the VU University Medical Center approved the study, and all participants gave written informed consent. The study was performed in accordance with the Declaration of Helsinki (2008) and the Dutch Medical Research involving Human Subjects Act (WMO). The trial was registered in the Dutch Trial Register (NTR5017). For this paper, the EMERGE were followed (De Geest et al., 2018).

### Study Population

Patients aged 45–75 years using antihypertensive medication (including beta-blockers, calcium antagonists, diuretics, angiotensin converting enzyme (ACE) inhibitors, and angiotensin II-receptor antagonists) for at least twelve months and indicating to have hypertension by self-report were eligible to participate. We excluded patients who had insufficient Dutch language skills or used medication-intake supporting services provided by the pharmacy, i.e., repeat dispensing and pill packaging.

Patients non-adherent according to pharmacy dispensing data were selected using the selection method of the SFK, which is developed by the Royal Dutch Pharmacists Association (SFK, 2013). SFK registers information on dispensed drugs and calculates the Proportion of Days Covered (PDC). Patients with a PDC < 80% for one antihypertensive drug class during the last six months were considered non-adherent (Nau, 2012). In each pharmacy a random sample of 75 patients was selected for inclusion using a randomization table. The selected patients were invited to participate and received the baseline questionnaire including the Medication Adherence Report Scale (MARS-5) (Horne, 2013). The MARS-5 assessed patients self-reported non-adherence to antihypertensive medication. Patients willing to participate were included if they were non-adherent based on both pharmacy dispensing data (PDC < 80%) and the self-report questionnaire (MARS-5 < 25). This study focused on medication adherence in the implementation phase of treatment (Vrijens et al., 2012).

### Intervention Group

Participants in the intervention group received the CATI intervention program in addition to usual care. The program was executed by the pharmacist and included two consultations in the pharmacy. Participating pharmacists received a training on how to deliver the intervention program.

#### First Consultation

The first consultation started with a semi-structured interview, called the QBS (Van der Laan et al., 2017). The aim of the QBS was to explore participants’ barriers to adhere to medication assessed with twelve questions. Based upon the identified barrier(s) at least one corresponding intervention module was selected according to the TIG (Van der Laan et al., 2017). The TIG contains an overview of intervention recommendations divided in five intervention modules: (1) Providing Information, (2) Providing Tools, (3) Dealing with Side Effects, (4) Overcoming Practical Problems, and (5) Diminishing Negative Beliefs. The first consultation continued with discussing participants’ illness and treatment representations and providing tailored information regarding potential risks of hypertension, use of medication and living a healthy lifestyle. Herewith, participants’ understanding of hypertension and the perceived need to be adherent to antihypertensive treatment would be increased, which is emphasized as important for achieving long-term medication adherence by the Common Sense Model of Self-regulation (Meyer et al., 1985). Subsequently, the pharmacist provided the participant with tailored recommendations from the selected intervention module to overcome the identified barriers. Finally, the participant received a written summary of the consultation including the information and recommendations provided.

#### Follow-Up Consultation

Approximately three months after the first consultation, a follow-up consultation was planned with the participant. The purpose of this follow-up consultation was to discuss participants’ implementation of and experiences with the discussed information and recommendations.

### Control Group

Participants in the control group only received usual care according to the Dutch guidelines of the Royal Dutch Pharmacists Association (KNMP, 2006). This care, usually delivered by a pharmacy technician, consist of checking and dispensing of prescribed drugs, providing instructions on medication use, and providing information about intended effects and possible side effects, during first and second dispensing. Blood pressure measurements in the control group were executed by the pharmacy technician.

### Outcome Measures

Both intervention and control group participants received a questionnaire by post at baseline and after three, six and nine months. This comprehensive questionnaire assessed medication adherence, beliefs about medicines, quality of life and illness perceptions.

The primary outcome was self-reported medication adherence assessed with MARS-5 (Horne, 2013). The MARS-5 comprises five statements of adherence-related behavior: “I alter the dose of my medicines,” “I stop taking my medicines for a while,” “I decide to miss out on a dose of my medicines,” “I forget to take my medicines,” and “I take less of my medicines than instructed.” Each statement is rated on a five-point scale, from 1 (always) to 5 (never). The MARS-5 sum score was calculated, ranging from 5 to 25 points where a higher score indicates better adherence. The MARS-5 sum score was also dichotomized, where a score below 25 points indicates non-adherence. The MARS-5 questionnaire is an easy-to-use tool and shows acceptable reliability and validity (Horne et al., 2001; Cohen et al., 2009; Mora et al., 2011; Bäck et al., 2012; Salt et al., 2012; Horne, 2013; Lin et al., 2018).

Secondary outcomes included participants’ beliefs about medicines assessed with the validated and reliable Specific Beliefs about Medicines Questionnaire (BMQ) (Horne and Weinman, 1999a; Horne et al., 1999b). The BMQ consists of ten questions scored on a 5-point Likert scale and is subdivided into a Necessity scale and Concern scale, both ranging from 5 to 25 points.

Participants’ quality of life was assessed with the validated and reliable 12-Item Short Form (SF-12) questionnaire (Hurst et al., 1998). The SF-12 questionnaire consists of twelve questions covering eight domains of health. The eight domains produces two summary scores including physical health (PCS) and mental health (MCS) both ranging from 0 to 100.

Participants’ illness perceptions were assessed using the reliable and validated Brief Illness Perceptions Questionnaire (Brief IPQ-9) (Broadbent et al., 2006). The IPQ-9 is a nine-item scale designed to assess participants’ cognitive and emotional representations of illness. All items except the causal question are rated on a 0 to 10 point scale.

Participants’ systolic and diastolic blood pressure was measured three times during the nine months follow-up according to a standardized protocol. In the intervention group the pharmacist performed the measurements at the first consultation, at the follow-up consultation and at a final pharmacy visit. For the control group the blood pressure was measured by a pharmacy technician during three additionally scheduled pharmacy visits. At each visit the blood pressure was measured three times, each two minutes apart, in seated position with an automatic sphygmomanometer (Lee et al., 2006). Measured blood pressure was calculated as the mean of the second and third measurement values.

### Sample Size

A sample size calculation for proportions, as described in the study protocol (Van der Laan et al., 2017), was performed on a clinical relevant difference of 20% in percentage of adherent participants on the self-reported MARS-5. Based on this calculation a total sample size of 156 participants was required.

### Statistical Analyses

Descriptive statistics were used to describe patient characteristics. Means and standard deviations for continuous variables and frequencies and percentages for categorical variables were presented. For non-normal continuous outcomes medians with interquartile ranges were also provided. To compare baseline values between the intervention and control group independent sample *t*-tests and chi-square tests were used. The effect analyses of the intervention program by comparing the differences between the intervention and control group were performed according to the “intention-to-treat” principle. Linear mixed-model analyses were used for continuous outcomes, and logistic generalized estimating equation (GEE) analyses were used for dichotomous outcomes. Logistic GEE analyses were preferred over logistic mixed-model analyses because of the instability of the latter and the overestimation of the effect estimates (Twisk, 2006). For all outcomes both an overall effect of the intervention program and effects of the intervention program at the different time points were estimated (three, six, and nine months). For each outcome variable crude regression coefficients were calculated (only adjusted for the baseline value of the particular outcome), as well as adjusted regression coefficients (adjusted for the baseline value of the particular outcome, the MARS-5 baseline score and possible confounders age, gender and education level). For the GEE analyses both crude and adjusted odds ratios (OR) were calculated. In addition to the main analyses, several sensitivity analyses were performed. First, a “per protocol” analyses was performed including all intervention participants that did visit the pharmacy for both the first and follow-up consultation. Second, several subgroup analyses were performed to stratify for gender, younger (45–55) and older (56–75) age and normal and high blood pressure measured at the first visit (baseline). Another subgroup analysis was performed including the intervention participants with ≥ 3 barriers identified with the QBS during the first consultation. A final subgroup analysis was performed using a more rigorous cut-off value for the primary outcome, including participants with a MARS-5 score of ≤ 23 at baseline (Mardby et al., 2007; Sjolander et al., 2013). A score of > 23 was considered adherent. Mixed-model and GEE analyses were performed using Stata/SE version 14.0 (StataCorp LP, College Station, TX, United States). All other analyses were performed using SPSS version 22.0 (IBM Corp, Armonk, NY, United States).

## Results

### Baseline Characteristics

**FIGURE 1:**
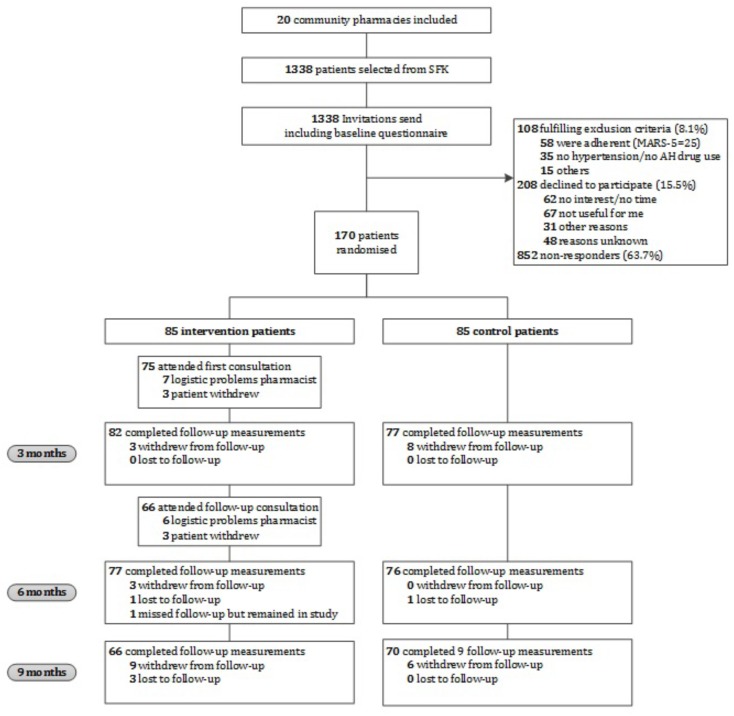
Flow chart of the CATI study participants. AH, antihypertensive; MARS-5, Medication Adherence Report Scale; SFK, Foundation for Pharmaceutical Statistics.

**Figure 1** provides an overview of the flow of participants through the study. In total, 170 participants were included (85 in the intervention group and 85 in the control group). At baseline, the MARS-5 score was 0.9 points lower in the intervention group as compared to the control group, which indicates that the intervention group was significantly less adherent than the control group (mean difference -0.9 [95% CI: -1.76 to -0.12], *p* = 0.024) (**Table 1**). No other relevant differences with respect to participant characteristics between the intervention and control group were found (**Table 1**).

**Table 1:** Baseline characteristics of participants of the CATI study.

	Total study population (*n* = 170)	Intervention group (*n* = 85)	Control group (*n* = 85)	*p*-value^c^
		
	N (%) or mean ± SD or median (P_25th_ to P_75th_)	N (%) or mean ± SD or median (P_25th_ to P_75th_)	N (%) or mean ± SD or median (P_25th_ to P_75th_)	
Female gender	86 (50.6)	44 (51.8)	42 (49.4)	0.759
Age	61.1 ± 7.9	60.3 ± 7.9	61.9 ± 7.9	0.208
**Origin** Dutch native Immigrant, Western Immigrant, non-Western	151 (88.8) 9 (5.3) 10 (5.9)	78 (91.8) 3 (3.5) 4 (4.7)	73 (85.9) 6 (7.1) 6 (7.1)	0.457
**Education level** Low Moderate High	43 (25.3) 66 (38.8) 61 (35.9)	23 (27.1) 31 (36.5) 31 (36.5)	20 (23.5) 35 (41.2) 30 (35.3)	0.791
**Employment status** Working	72 (42.4)	38 (44.7)	34 (40.0)	0.535
**Living situation** Alone	28 (16.5)	10 (11.8)	18 (21.2)	0.098
**Alcohol use** Yes	118 (69.4)	59 (69.4)	59 (69.4)	1.000
**Tobacco use** Yes	15 (8.8)	6 (7.1)	9 (10.6)	0.417
No. of cardiovascular diseases	1.9 ± 1.0	1.9 ± 1.1	1.9 ± 0.9	0.765
No. of total prescribed medicines	4.1 ± 2.6	4.0 ± 2.7	4.1 ± 2.4	0.811
No. of antihypertensive medicines	1.8 ± 0.8	1.8 ± 0.8	1.8 ± 0.8	0.927
**Antihypertensive medicines** Beta blockers Calcium antagonists Diuretics ACE inhibitors / angiotensin II-receptor antagonists	59 (34.7) 54 (31.8) 56 (32.9) 124 (72.9)	30 (35.3) 28 (32.9) 27 (31.8) 60 (70.6)	29 (34.1) 26 (30.6) 29 (34.1) 64 (75.3)	0.872 0.742 0.744 0.490
**Blood pressure (mmHg)^a^** Systolic Diastolic	143.2 ± 17.6 86.7 ± 12.7	145.3 ± 16.0 88.0 ± 12.5	140.8 ± 19.0 85.2 ± 12.9	0.131 0.191
**High blood pressure^a,b^** Yes	80 (57.1)	46 (63.0)	34 (50.7)	0.143
**Medication adherence (MARS-5)**				
Sum score (5–25)	22.1 ± 2.7 23 (21 to 24)	21.6 ± 3.1 23 (21.0 to 24.0)	22.5 ± 2.2 23.0 (22.0 to 24.0)	0.024^∗^
**Quality of life (SF-12)** PCS (0–100)	46.3 ± 10.1 49.9 (39.2 to 54.2)	45.7 ± 10.2 49.3 (38.7 to 53.9)	46.9 ± 10.0 50.8 (42.2 to 54.2)	0.438
MCS (0–100)	52.7 ± 8.3 55.4 (49.7 to 57.9)	53.1 ± 7.7 55.2 (48.9 to 58.6)	52.3 ± 8.9 55.7 (50.7 to 57.7)	0.508
**Beliefs about medicines (BMQ)**				
Necessity (5–25)	15.6 ± 4.2	15.6 ± 4.4	15.7 ± 4.1	0.813
Concern (5–25)	12.6 ± 3.7	12.9 ± 3.7	12.3 ± 3.7	0.277
**Illness Perceptions (IPQ-9)** Consequences (0–10)	3.3 ± 2.8 3.0 (1.0 to 6.0)	3.7 ± 2.8 3.0 (1.0 to 6.0)	2.9 ± 2.7 2.0 (0.0 to 5.0)	0.083
Timeline (0–10)	8.2 ± 2.5 9.0 (7.0 to 10.0)	8.4 ± 2.3 10.0 (7.0 to 10.0)	8.1 ± 2.7 9.0 (7.0 to 10.0)	0.417
Personal control (0–10)	5.2 ± 2.4	5.0 ± 2.5	5.3 ± 2.3	0.423
Treatment control (0–10)	7.4 ± 1.9 8.0 (7.0 to 9.0)	7.4 ± 1.8 8.0 (7.0 to 9.0)	7.4 ± 1.9 7.0 (6.5 to 9.0)	0.986
Identity (0–10)	3.2 ± 2.8 3.0 (0.0 to 5.25)	3.5 ± 2.8 3.0 (1.0 to 6.0)	2.9 ± 2.7 2.0 (0.0 to 5.0)	0.133
Illness concerns (0–10)	4.7 ± 2.8	4.7 ± 2.9	4.7 ± 2.8	0.978
Coherence (0–10)	6.0 ± 2.7	5.9 ± 3.0	6.1 ± 2.4	0.548
Emotional representation (0–10)	2.7 ± 2.7 2.0 (0.0 to 5.0)	2.7 ± 2.5 2.0 (0.0 to 5.0)	2.6 ± 2.9 2.0 (0.0 to 4.0)	0.842


*Abbreviations: BMQ, Specific Beliefs about Medicines Questionnaire; IPQ-9, Illness Perceptions Questionnaire; MARS-5, Medication Adherence Report Scale; MCS, Mental Component Score; PCS, Physical Component Score; SD, standard deviation; SF-12, 12-item Short Form. ^*a*^Data from 30 participants is missing. ^*b*^High blood pressure is defined as an elevated blood pressure exceeding 140 mm Hg in systolic phase and or 90 mm Hg in diastolic phase. ^*c*^Independent sample *t*-tests and chi-square tests were used to test for differences between the intervention and control group. ^∗^Statistically significant.*

### Declined to Participate and Non-responders

In total, 1338 patients were invited to participate. Of them, 108 (8.1%) did not meet the inclusion criteria, 208 (15.5%) declined to participate, and 852 (63.7%) did not respond (**Figure 1**). Patients who declined to participate did not differ in gender from participants, however, they were significantly older (*p* ≤ 0.001) and had a lower level of education (*p* = 0.005). Reasons indicated for non-participation were no interest, no time, or not needed according to the patient. Gender and age of non-responders were comparable to participants.

### Intervention Program

The first consultation of the intervention program was held with 75 participants (88.2%) and the follow-up consultation with 66 participants (77.6%). The average period between the first and follow-up consultation was 94 days. In most cases missed consultations were due to logistic and time management problems of pharmacists. The average time of the first and follow-up consultation was 36 min (range: 15–85 min) and 20 min (range: 5–45 min), respectively. Participants who did not attend both consultations did not differ in age, gender, origin, education level, employment status or living situation from participants who did attend both consultations, however, they used significantly more antihypertensive medicines (*p* = 0.044).

### Primary Outcome

**Table 2A** presents the mean scores and proportions of the primary outcome. In the “intention-to-treat” analysis, no significant differences were found in self-reported adherence over time or after three, six and nine months between the intervention and control group (**Table 3A**). Results of the sensitivity analyses are presented in **Appendix A**. Per protocol and most subgroup analyses did not show different results. However, in the subgroup analysis in which we only included participants with ≥ 3 barriers identified during the first consultation, a significant intervention effect was found on self-reported medication adherence after nine months (mean difference 0.84 [95% CI: 0.03 to 1.65], *p* = 0.042). In the subgroup analysis where we used a more rigorous cut-off value for the MARS-5 score, a more positive intervention effect was found (mean difference 0.43 [95% CI: -0.30 to 1.15]), however, not statistically significant.

**Table 2A:** Mean scores ± SD and proportions (%) of the primary and secondary outcomes at each measurement point.

		Baseline		T1		T2		T3	
		Mean ± SD	*n*	Mean ± SD	*n*	Mean ± SD	*n*	Mean ± SD	*n*
**Primary outcome**								
MARS-5 sum score	Intervention	21.6 ± 3.1	85	22.5 ± 2.5	82	22.7 ± 2.4	77	22.8 ± 2.3	66
	Control	22.5 ± 2.2	85	23.0 ± 1.9	77	22.9 ± 2.2	76	23.1 ± 2.3	70

		***n (%)***	***n***	***n (%)***	***n***	***n (%)***	***n***	***n (%)***	***n***

MARS-5 (< 25)	Intervention	85 (100)	85	71 (86.6)	82	65 (84.4)	77	57 (86.4)	66
	Control	85 (100)	85	64 (83.1)	77	65 (85.5)	76	55 (78.6)	70

		**Mean ± SD**	***n***	**Mean ± SD**	***n***	**Mean ± SD**	***n***	**Mean ± SD**	***n***

**Secondary outcomes**								
SF-12 PCS (0–100)	Intervention	45.7 ± 10.2	85	45.5 ± 9.6	82	46.0 ± 10.3	77	46.0 ± 10.2	66
	Control	46.9 ± 10.0	85	46.6 ± 8.8	77	46.5 ± 9.0	74	46.3 ± 9.7	70
SF-12 MCS (0–100)	Intervention	53.1 ± 7.7	85	53.2 ± 7.5	82	52.9 ± 8.0	77	52.9 ± 8.3	66
	Control	52.3 ± 8.9	85	53.6 ± 8.0	77	53.0 ± 8.4	74	52.5 ± 8.7	70
BMQ Necessity (5–25)	Intervention	15.6 ± 4.4	85	15.8 ± 4.3	82	16.2 ± 4.5	77	16.9 ± 4.4	66
	Control	15.7 ± 4.1	85	15.7 ± 4.0	77	16.1 ± 4.5	76	16.0 ± 4.2	70
BMC Concern (5–25)	Intervention	12.9 ± 3.7	85	13.1 ± 3.9	82	12.5 ± 4.1	77	12.6 ± 3.9	66
	Control	12.3 ± 3.7	85	12.7 ± 3.9	77	12.3 ± 4.4	76	12.4 ± 3.9	70
IPQ-9 Consequences (0–10)	Intervention	3.7 ± 2.8	85	3.3 ± 2.5	82	3.5 ± 2.7	77	3.7 ± 2.8	66
	Control	2.9 ± 2.7	85	3.3 ± 2.6	77	3.5 ± 2.7	76	3.4 ± 2.8	70
IPQ-9 Timeline (0–10)	Intervention	8.4 ± 2.3	84	8.5 ± 2.0	81	8.4 ± 2.3	77	8.5 ± 2.4	66
	Control	8.1 ± 2.7	85	8.1 ± 2.5	75	7.9 ± 2.6	76	8.2 ± 2.4	69
IPQ-9 Personal control (0–10)	Intervention	5.0 ± 2.5	85	5.6 ± 2.3	81	5.4 ± 2.4	77	5.6 ± 2.3	66
	Control	5.3 ± 2.3	85	5.8 ± 2.3	76	5.8 ± 2.4	76	5.9 ± 2.3	69
IPQ-9 Treatment control (0–10)	Intervention	7.4 ± 1.8	84	7.5 ± 2.0	81	7.4 ± 2.2	77	7.6 ± 1.8	66
	Control	7.4 ± 1.9	85	7.6 ± 1.7	75	7.4 ± 1.9	76	7.8 ± 1.6	69
IPQ-9 Identity (0–10)	Intervention	3.5 ± 2.8	85	3.2 ± 2.7	82	3.2 ± 2.7	77	3.2 ± 2.6	66
	Control	2.9 ± 2.7	85	2.9 ± 2.7	77	3.1 ± 2.7	76	3.2 ± 2.9	70
IPQ-9 Illness concerns (0–10)	Intervention	4.7 ± 2.9	85	4.8 ± 2.9	81	4.7 ± 2.7	77	4.8 ± 2.9	66
	Control	4.7 ± 2.8	85	4.5 ± 2.6	76	4.2 ± 2.9	76	4.3 ± 2.7	70
IPQ-9 Coherence (0–10)	Intervention	5.9 ± 3.0	85	6.2 ± 2.6	82	6.3 ± 2.8	77	6.3 ± 2.7	66
	Control	6.1 ± 2.4	85	6.2 ± 2.7	76	5.8 ± 2.7	76	6.4 ± 2.4	69
IPQ-9 Emotional representation (0–10)	Intervention	2.7 ± 2.5	85	2.6 ± 2.4	81	2.7 ± 2.7	77	3.3 ± 2.9	66
	Control	2.6 ± 2.9	85	2.6 ± 2.5	76	2.9 ± 2.9	76	2.9 ± 2.8	70


*BMQ, Specific Beliefs about Medicines Questionnaire; IPQ-9, Illness Perceptions Questionnaire; MARS-5, Medication Adherence Report Scale; MCS, Mental Component Score; PCS, Physical Component Score; SD, standard deviation, SF-12, 12-item Short Form.*

**Table 2B:** Mean scores ± SD of systolic and diastolic blood pressure measurements at three visits during study period.

		Visit 1 (baseline)		Visit 2		Visit 3	
		Mean ± SD	*n*	Mean ± SD	*n*	Mean ± SD	*n*
**Secondary outcomes**							
Systolic blood pressure	Intervention	145.3 ± 16.0	73	142.9 ± 19.0	66	145.1 ± 16.7	55
	Control	140.8 ± 19.0	67	140.0 ± 17.8	65	142.9 ± 17.4	55
Diastolic blood pressure	Intervention	88.0 ± 12.5	73	88.1 ± 13.9	66	87.3 ± 13.6	55
	Control	85.2 ± 12.9	67	85.6 ± 12.5	65	86.7 ± 12.2	55


*SD, standard deviation.*

**Table 3A:** Intervention effects from linear mixed model and logistic GEE analyses of the primary outcome and secondary outcomes.

	Overall effect		T1		T2		T3	
	Difference (95% CI)	*p*-value	Difference (95% CI)	*p*-value	Difference (95% CI)	*p*-value	Difference (95% CI)	*p*-value
**Primary outcome**								
MARS-5 sum score								
Crude^a^	0.17 (–0.29 to 0.62)	0.472	–0.05 (–0.59 to 0.48)	0.851	0.34 (–0.20 to 0.88)	0.220	0.27 (–0.30 to 0.83)	0.354
Adjusted^b^	0.18 (–0.27 to 0.63)	0.440	–0.04 (–0.57 to 0.49)	0.874	0.35 (–0.18 to 0.89)	0.198	0.27 (–0.28 to 0.83)	0.337

	**OR (95% CI)**	***p-value***	***OR (95% CI)***	***p-value***	***OR (95% CI)***	***p-value***	***OR (95% CI)***	***p-value***

MARS-5 (<25)								
Crude	1.03 (0.94 to 1.13)	0.467	1.04 (0.93 to 1.16)	0.543	0.99 (0.88 to 1.10)	0.812	1.09 (0.96 to 1.24)	0.179
Cdjusted	1.03 (0.94 to 1.13)	0.505	1.03 (0.92 to 1.16)	0.577	0.98 (0.88 to 1.10)	0.765	1.09 (0.96 to 1.23)	0.190

	**Difference (95% CI)**	***p*-value**	**Difference (95% CI)**	***p*-value**	**Difference (95% CI)**	***p*-value**	**Difference (95% CI)**	***p*-value**

**Secondary outcomes**								
SF-12 PCS								
Crude	–0.41 (–2.08 to 1.26)	0.631	–0.61 (–2.64 to 1.43)	0.558	–0.28 (–2.35 to 1.80)	0.794	–0.33 (–2.48 to 1.82)	0.764
Adjusted	–0.10 (–1.77 to 1.58)	0.911	–0.29 (–2.33 to 1.75)	0.781	0.04 (–2.04 to 2.11)	0.971	–0.02 (–2.18 to 2.13)	0.984
SF-12 MCS								
Crude	–0.55 (–2.33 to 1.22)	0.541	–0.94 (–3.04 to 1.15)	0.378	–0.54 (–2.67 to 1.60)	0.622	–0.15 (–2.35 to 2.10)	0.896
Adjusted	–0.84 (–2.63 to 0.96)	0.362	–1.22 (–3.34 to 0.89)	0.258	–0.81 (–2.96 to 1.34)	0.460	–0.45 (–2.67 to 1.78)	0.695
BMQ Necessity								
Crude	0.68 (–0.03 to 1.38)	0.060	0.40 (–0.52 to 1.31)	0.393	0.54 (–0.38 to 1.47)	0.250	1.23 (0.25 to 2.20)	0.013^∗^
Adjusted	0.70 (–0.01 to 1.42)	0.054	0.43 (–0.50 to 1.35)	0.366	0.57 (–0.36 to 1.51)	0.230	1.25 (0.27 to 2.24)	0.012^∗^
BMQ Concern								
Crude	–0.11 (–0.88 to 0.65)	0.773	0.03 (–0.87 to 0.94)	0.944	–0.11 (–1.02 to 0.81)	0.818	–0.35 (–1.30 to 0.60)	0.465
Adjusted	–0.12 (–0.90 to 0.65)	0.755	0.02 (–0.89 to 0.93)	0.963	–0.12 (–1.04 to 0.81)	0.804	–0.36 (–1.32 to 0.59)	0.455
IPQ-9 Consequences								
Crude	–0.29 (–0.80 to 0.22)	0.271	–0.31 (–0.93 to 0.31)	0.331	–0.34 (–0.98 to 0.29)	0.288	–0.17 (–0.83 to 0.49)	0.612
Adjusted	–0.34 (–0.85 to 0.17)	0.187	–0.36 (–0.98 to 0.26)	0.257	–0.40 (–1.03 to 0.23)	0.217	–0.23 (–0.89 to 0.42)	0.488
IPQ-9 Timeline								
Crude	0.28 (–0.22 to 0.77)	0.270	0.23 (–0.40 to 0.86)	0.470	0.31 (–0.32 to 0.94)	0.336	0.29 (–0.37 to 0.96)	0.386
Adjusted	0.27 (–0.24 to 0.77)	0.298	0.22 (–0.42 to 0.86)	0.497	0.30 (–0.34 to 0.94)	0.358	0.29 (–0.39 to 0.96)	0.404
IPQ-9 Personal control								
Crude	–0.03 (–0.53 to 0.46)	0.893	0.00 (–0.65 to 0.65)	0.997	–0.15 (–0.80 to 0.51)	0.655	0.07 (–0.62 to 0.76)	0.835
Adjusted	–0.06 (–0.56 to 0.44)	0.815	–0.03 (–0.68 to 0.63)	0.937	–0.18 (–0.84 to 0.49)	0.602	0.05 (–0.65 to 0.75)	0.887
IPQ-9 Treatment control								
Crude	0.08 (–0.33 to 0.49)	0.701	0.06 (–0.44 to 0.57)	0.800	0.20 (–0.31 to 0.71)	0.440	–0.04 (–0.57 to 0.49)	0.892
Adjusted	0.09 (–0.32 to 0.50)	0.669	0.08 (–0.43 to 0.58)	0.766	0.21 (–0.30 to 0.72)	0.421	–0.03 (–0.56 to 0.51)	0.923
IPQ-9 Identity								
Crude	–0.20 (–0.70 to 0.30)	0.427	–0.12 (–0.71 to 0.48)	0.700	–0.18 (–0.78 to 0.42)	0.566	–0.33 (–0.95 to 0.30)	0.302
Adjusted	–0.26 (–0.75 to 0.23)	0.293	–0.18 (–0.77 to 0.41)	0.551	–0.23 (–0.83 to 0.36)	0.439	–0.39 (–1.01 to 0.23)	0.214
IPQ-9 Illness concerns								
Crude	0.39 (–0.17 to 0.95)	0.174	0.23 (–0.43 to 0.90)	0.497	0.49 (–0.18 to 1.16)	0.154	0.45 (–0.24 to 1.15)	0.202
Adjusted	0.30 (–0.26 to 0.85)	0.296	0.14 (–0.52 to 0.80)	0.681	0.40 (–0.27 to 1.10)	0.245	0.36 (–0.34 to 1.05)	0.312
IPQ-9 Coherence								
Crude	0.32 (–0.24 to 0.87)	0.265	0.16 (–0.55 to 0.87)	0.654	0.76 (0.04 to 1.48)	0.040^∗^	–0.01 (–0.77 to 0.75)	0.986
Adjusted	0.34 (–0.20 to 0.89)	0.220	0.19 (–0.51 to 0.90)	0.591	0.78 (0.06 to 1.49)	0.034^∗^	0.03 (–0.72 to 0.79)	0.929
IPQ-9 Emotional representation								
Crude	–0.09 (–0.58 to 0.40)	0.717	–0.17 (–0.77 to 0.44)	0.587	–0.26 (–0.87 to 0.35)	0.406	0.24 (–0.39 to 0.88)	0.454
Adjusted	–0.12 (–0.61 to 0.37)	0.620	–0.20 (–0.81 to 0.40)	0.511	–0.29 (–0.91 to 0.32)	0.347	0.22 (–0.42 to 0.86)	0.509


*BMQ, Specific Beliefs about Medicines Questionnaire; CI, confidence interval; GEE, Generalized Estimating Equation; IPQ-9, Illness Perceptions Questionnaire; MARS-5, Medication Adherence Report Scale; MCS, Mental Component Score; OR, Odds Ratio; PCS, Physical Component Score; SF-12, 12-item Short Form. ^*a*^Corrected for baseline value of the particular outcome. ^*b*^Additionally corrected for MARS-5 score at baseline and possible confounders age, gender and education level. ^∗^Statistically significant.*

**Table 3B:** Intervention effects from linear mixed model of the clinical secondary outcome.

	Overall effect		Visit 2		Visit 3	
	Difference (95% CI)	*p*-value	Difference (95% CI)	*p*-value	Difference (95% CI)	*p*-value
**Systolic blood pressure**						
Crude^a^	1.32 (–2.83 to 5.47)	0.533	2.14 (–2.78 to 7.07)	0.394	0.30 (–5.03 to 5.62)	0.913
Adjusted^b^	1.52 (–2.71 to 5.75)	0.481	2.30 (–2.69 to 7.29)	0.366	0.56 (–4.83 to 5.96)	0.838
**Diastolic blood pressure**						
Crude	0.93 (–2.30 to 4.15)	0.573	1.79 (–1.90 to 5.48)	0.342	–0.17 (–4.13 to 3.79)	0.932
Adjusted	0.63 (–2.65 to 3.92)	0.706	1.49 (–2.25 to 5.23)	0.435	–0.46 (–4.47 to 3.55)	0.822


*CI, confidence interval. ^a^Corrected for baseline value of the particular outcome. ^*b*^Additionally corrected for MARS-5 score at baseline and possible confounders age, gender and education level.*

### Secondary Outcomes

In **Tables 2A**,**B** the mean scores of the secondary outcomes are listed. For the quality of life (SF-12), the illness perceptions (IPQ-9) and the systolic and diastolic blood pressure, no significant differences over time or at the different time points between the intervention and control group were found (**Tables 3A**,**B**). For the beliefs about medicines, a borderline significant difference over time was found on the BMQ Necessity scale (**Tables 3A**,**B**). This overall effect was found because participants in the intervention group had a significantly higher BMQ Necessity score at nine months follow-up (mean difference 1.25 [95% CI: 0.27 to 2.24], *p* = 0.012). This means that they had significantly stronger beliefs about the necessity of using their medicines as compared to the control group.

## Discussion

The aim of this study was to evaluate the effectiveness of the CATI intervention program to enhance self-reported adherence to antihypertensive medication as compared to usual care. There were no statistically significant effects in adherence-related behavior, quality of life, illness perceptions, beliefs about medicines (concern scale), and blood pressure. For the beliefs about medicines, we did find a statistical significant intervention effect on the BMQ Necessity scale after nine months.

A systematic review of Gwadry-Sridhar et al. (2013) reported that only 35.1% of comparable intervention studies aimed to directly improve adherence in patients with hypertension did significantly improve medication adherence. In studies with quite comparable intervention programs inconsistent findings were reported (Morgado et al., 2011; Stewart et al., 2014; Nguyen et al., 2016). For example, in a study of Morgado et al. (2011) a pharmaceutical care program including two counseling visits to the pharmacy in patients using antihypertensive medication was evaluated. They reported statistically significant effects on both blood pressure control and self-reported medication adherence at nine months. In a study of Stewart et al. (2014) patients visited the pharmacy three times and received a package of interventions, including motivational interviewing, medication review and prescription refill reminders. They concluded that the intervention had a significant effect on blood pressure, but not on medication adherence. In a study of Nguyen et al. (2016) a multifaceted medication management intervention for patients with hypertension, type 2 diabetes or other cardiovascular disease significantly improved medication adherence. This study also found that intervention patients had significantly stronger necessity beliefs compared to control patients. This latter result is in line with the finding of our study.

There are several possible explanations for the absence of effects on medication adherence-related behavior in our study. First, the design of our study was a parallel-group randomized controlled trial in which individuals within one pharmacy were randomized into the intervention or control group. It might have been better to apply cluster randomization to control for contamination across groups. However, to avoid contamination in our study the intervention group was executed by the pharmacist, whereas the control group was executed by the pharmacy technician. Second, since large numbers of patients did not respond (63.7%) and declined to participate (15.5%) we cannot rule out selection bias. It might be possible that we missed patients that were unaware of their incompetence to properly use their medicines and therefore did not score non-adherent on the self-reported questionnaire. In addition, patients with strong negative beliefs toward medicines are probably not responsive for participation in intervention studies, while these patients are the population most interesting to target. With respect to drop-out, participants who did not attend the consultations used significantly more antihypertensive medicines compared to participants who did attend both consultations. This can be seen as a limitation, since patients with multiple medicines will benefit most from counseling interventions. However, we consider the bias of study results limited because in most cases the reason for not performing a consultation were logistic and time management problems of pharmacists. Third, the absence of effects could be related to the eligibility criteria of the study population, especially with regard to the outcome measure. There has been some debate about the appropriateness of MARS-5 as a medication adherence measure in the literature (van de Steeg et al., 2009; Tommelein et al., 2014). An explanation for these inconsistent results could be the use of pharmacy refill data as reference standard, since this measures another underlying construct compared to self-reported questionnaires. However, a large number of studies have shown acceptable validity and reliability of MARS-5 and therefore we have chosen this measure for this study (Cohen et al., 2009; Mora et al., 2011; Bäck et al., 2012; Salt et al., 2012; Horne, 2013; Lin et al., 2018). The chosen cut-off value for MARS-5 might be a plausible explanation for the absence of effects. A self-reported MARS-5 score of below 25 points, indicates marginally non-adherent behavior. This resulted in too little room for improvement. Results of the sensitivity analysis on participants with lower self-reported adherence at baseline (MARS-5 ≤ 23) were more positive than the main analysis. It may, therefore, be more efficient to target an intervention like this on patient groups with a lower degree of adherence. Another outcome measure limitation included that we were not able to collect pharmacy refill data after baseline. Because if a participant started repeat dispensing (medication-intake supporting service of pharmacy) during the study period, the dispensing was regulated and therefore participants could no longer be identified as non-adherent with the SFK selection method. Since pharmacy refill data could not be collected, we were not able to combine two different methods to assess medication adherence, a strategy that has been suggested in the literature (Sabate, 2003; Lam and Fresco, 2015). A patient group who experiences multiple barriers to adhere to medication and therefore might deal with more structural adherence problems, might also be more eligible for this kind of intervention. This is confirmed by the more positive and significant intervention effects in the sensitivity analyses on participants with ≥ 3 identified barriers. Since only 57% of participants had an uncontrolled blood pressure at baseline, using uncontrolled blood pressure as an eligibility criterion in adherence-enhancing interventions must be considered. However, it might also be important to target both patients with or without uncontrolled blood pressure, since non-adherent behavior is continuous and dynamic and can occur over time (Sabate, 2003). Fourth, since we only found a significant effect in the main analyses on the BMQ Necessity scale after nine months, it might be possible that improvement of actual adherence-related behavior can only be expected after a longer period of time (Horne and Weinman, 1999a; Ross et al., 2004; Rajpura and Nayak, 2014). In the literature the Stages of Change model describes that health-related behavior change is rarely easy and requires a gradual progression of small steps (Prochaska and Velicer, 1997). In early stages, becoming more aware of the need for treatment, is an important factor for the beginning of small changes in behavior. It might, therefore, be more efficient to target patients in the initiation phase of medicine-taking (at the start of therapy). For instance, a study testing a pharmacist telephone counseling intervention found improvements in medication adherence in patients initiating therapy (Kooij et al., 2016). In contrast, it might also be efficient to target patients with long-term medication use, since these patients might experience treatment fatigue and therefore are at risk for medication non-adherence. Finally, since no effects were found on participants’ illness perceptions, the use of the Common Sense Model of Self-regulation as a foundation of adherence-enhancing interventions must be reconsidered.

### Implications for Practice and Research

Based on the results, we do not recommend to implement the CATI intervention program in the current form for the population selected in this study. The chosen cut-off value of MARS-5 might have been too liberal resulting in including participants with only marginally non-adherent behavior. Future studies should focus on how to select eligible patient groups with appropriate measures in order to effectively target adherence-enhancing interventions. Study populations with more severe non-adherent behavior and more barriers to adhere to medication, might benefit more from interventions, which is also confirmed by the more positive results of our sensitivity analyses. Future studies should also explore whether uncontrolled blood pressure should be added as an eligibility criterion. Moreover, future research might focus more on raising awareness and patients’ necessity beliefs of using medicines, in order to change patients’ adherence-related behavior. Finally, a process evaluation study would provide more insight into whether the ineffectiveness of the CATI intervention program was due to poor implementation or inadequacies of the intervention itself.

## Author Contributions

DvdL developed the study protocol, drafted the manuscript, coordinated data collection, analyzed the data, and reported the study results. JH and CB developed the study protocol, revised the manuscript, supported in data collection, analyzed the data, and reported the study results. PE, GN, and LvD participated in the design of the study and revised the manuscript. All authors read and approved the final manuscript.

## Conflict of Interest Statement

The authors declare that the research was conducted in the absence of any commercial or financial relationships that could be construed as a potential conflict of interest.

## Abbreviations

CATI: cardiovascular medication non-adherence tailored intervention
EMERGE: ESPACOMP medication adherence reporting guidelines
QBS: quick barrier scan
SFK: foundation for pharmaceutical statistics
TIG: tailored intervention guide
