# Supplementary material for: Effectiveness of a Patient-Tailored, Pharmacist-Led Intervention Program to Enhance Adherence to Antihypertensive Medication: The CATI Study
Source: Front Pharmacol. 2018 Sep 26;9:1057. doi: 10.3389/fphar.2018.01057 (PMC6169131; doi:10.3389/fphar.2018.01057)
Supplement: Supplementary file 1 [file Table_1.pdf]

APPENDIX A – Results of the sensitivity analyses for the primary outcome

Table A1 Per protocol analyses including intervention participants who attended both the first and follow-up consultation (N=151)

	**Overall effect**		**T1**		**T2**		**T3**	
	Difference (95% CI)	*p-value*	*Difference (95% CI)*	*p-value*	*Difference (95% CI)*	*p-value*	*Difference (95% CI)*	*p-value*
**MARS-5 sum score**								
crude [a]	0.08 (-0.38 to 0.54)	0.747	-0.18 (-0.73 to 0.37)	0.518	0.26 (-0.30 to 0.81)	0.365	0.20 (-0.40 to 0.76)	0.502
adjusted [b]	0.09 (-0.37 to 0.54)	0.706	-0.17 (-0.72 to 0.37)	0.537	0.27 (-0.28 to 0.82)	0.332	0.21 (-0.36 to 0.78)	0.473
	OR (95% CI)	*p-value*	*OR (95% CI)*	*p-value*	*OR (95% CI)*	*p-value*	*OR (95% CI)*	*p-value*
**MARS-5 (<25)**								
crude	1.05 (0.96 to 1.15)	0.317	1.05 (0.93 to 1.18)	0.418	0.99 (0.88 to 1.12)	0.878	1.12 (0.99 to 1.27)	0.071
adjusted	1.05 (0.95 to 1.15)	0.358	1.05 (0.93 to 1.18)	0.451	0.99 (0.88 to 1.11)	0.824	1.12 (099 to 1.27)	0.081

***Abbreviation** CI: confidence interval, MARS-5: Medication Adherence Report Scale*

[a] *Corrected for baseline value of the particular outcome*

[b] *Additionally corrected for possible confounders age, gender and education level*

Table A2 Subgroup analyses to stratify for gender

	Overall effect				T1				T2				T3			
	**Male gender**		**Female gender**		**Male gender**		**Female gender**		**Male gender**		**Female gender**		**Male gender**		**Female gender**	
	Difference (95% CI)	*p-value*	*Difference (95% CI)*	*p-value*	*Difference (95% CI)*	*p-value*	*Difference (95% CI)*	*p-value*	*Difference (95% CI)*	*p-value*	*Difference (95% CI)*	*p-value*	*Difference (95% CI)*	*p-value*	*Difference (95% CI)*	*p-value*
**MARS-5 sum score**																
crude [a]	0.29 (-0.28 to 0.86)	0.316	0.19 (-0.50 to 0.87)	0.596	0.16 (-0.54 to 0.87)	0.651	-0.12 (-0.90 to 0.66)	0.764	0.55 (-0.17 to 1.26)	0.132	0.28 (-0.52 to 1.07)	0.498	0.16 (-0.58 to 0.89)	0.678	0.54 (-0.29 to 1.36)	0.202
adjusted [b]	0.23 (-0.33 to 0.79)	0.417	0.24 (-0.46 to 0.94)	0.498	0.10 (-0.60 to 0.80)	0.780	-0.06 (-0.85 to 0.73)	0.874	0.49 (-0.22 to 1.19)	0.175	0.34 (-0.47 to 1.14)	0.412	0.10 (-0.63 to 0.83)	0.790	0.59 (-0.24 to 1.42)	0.164
	OR (95% CI)	*p-value*	*OR (95% CI)*	*p-value*	*OR (95% CI)*	*p-value*	*OR (95% CI)*	*p-value*	*OR (95% CI)*	*p-value*	*OR (95% CI)*	*p-value*	*OR (95% CI)*	*p-value*	*OR (95% CI)*	*p-value*
**MARS-5 (<25)**																
crude	1.00 (0.87 to 1.14)	0.953	1.07 (0.95 to 1.22)	0.276	1.06 (0.91 to 1.23)	0.473	1.02 (0.86 to 1.20)	0.842	0.92 (0.78 to 1.09)	0.352	1.05 (0.91 to 1.22)	0.507	1.01 (0.84 to 1.21)	0.900	1.18 (0.99 to 1.40)	0.066
adjusted	1.00 (0.88 to 1.14)	0.955	1.06 (0.94 to 1.20)	0.349	1.07 (0.92 to 1.24)	0.413	1.01 (0.85 to 1.19)	0.940	0.93 (0.79 to 1.10)	0.392	1.04 (0.90 to 1.20)	0.601	1.02 (0.85 to 1.22)	0.831	1.17 (0.98 to 1.38)	0.084

Abbreviation *CI: confidence interval, MARS-5: Medication Adherence Report Scale*

[a] *Corrected for baseline value of the particular outcome*

[b] *Additionally corrected for possible confounders age, gender and education level*

Table A3 Subgroup analyses to stratify for age

	Overall effect				T1				T2				T3			
	**Younger (45-55)**		**Older (56-75)**		**Younger (45-55)**		**Older (56-75)**		**Younger (45-55)**		**Older (56-75)**		**Younger (45-55)**		**Older (56-75)**	
	Difference (95% CI)	*p-value*	*Difference (95% CI)*	*p-value*	*Difference (95% CI)*	*p-value*	*Difference (95% CI)*	*p-value*	*Difference (95% CI)*	*p-value*	*Difference (95% CI)*	*p-value*	*Difference (95% CI)*	*p-value*	*Difference (95% CI)*	*p-value*
**MARS-5 sum score**																
crude [a]	0.24 (-0.84 to 1.32)	0.657	0.22 (-0.22 to 0.66)	0.324	-0.27 (-1.47 to 0.92)	0.653	0.09 (-0.46 to 0.64)	0.751	0.98 (-0.22 to 2.18)	0.110	0.24 (-0.32 to 0.79)	0.409	0.07 (-1.15 to 1.29)	0.910	0.41 (-0.18 to 1.00)	0.173
adjusted [b]	0.30 (-0.74 to 1.34)	0.570	0.21 (-0.21 to 0.64)	0.332	-0.27 (-1.45 to 0.91)	0.658	0.07 (-0.46 to 0.61)	0.787	0.98 (-0.21 to 2.17)	0.107	0.21 (-0.33 to 0.76)	0.445	0.07 (-1.14 to 1.29)	0.906	0.38 (-0.20 to 0.96)	0.195
	OR (95% CI)	*p-value*	*OR (95% CI)*	*p-value*	*OR (95% CI)*	*p-value*	*OR (95% CI)*	*p-value*	*OR (95% CI)*	*p-value*	*OR (95% CI)*	*p-value*	*OR (95% CI)*	*p-value*	*OR (95% CI)*	*p-value*
**MARS-5 (<25)**																
crude	1.00 (0.89 to 1.13)	0.977	1.03 (0.92 to 1.16)	0.573	1.00 (0.82 to 1.21)	0.965	1.04 (0.91 to 1.20)	0.529	0.94 (0.80 to 1.11)	0.487	0.99 (0.86 to 1.14)	0.904	1.07 (0.95 to 1.21)	0.294	1.06 (0.90 to 1.25)	0.473
adjusted	1.00 (0.91 to 1.12)	0.891	1.04 (0.92 to 1.17)	0.533	1.02 (0.86 to 1.22)	0.798	1.05 (0.91 to 1.21)	0.490	0.97 (0.84 to 1.12)	0.653	1.00 (0.87 to 1.15)	0.976	1.10 (0.97 to 1.24)	0.141	1.07 (0.91 to 1.26)	0.428

***Abbreviation** CI: confidence interval, MARS-5: Medication Adherence Report Scale*

[a] *Corrected for baseline value of the particular outcome*

[b] *Additionally corrected for possible confounders age, gender and education level*

Table A4 Subgroup analyses to stratify for blood pressure

	**Overall effect**				**T1**				**T2**				**T3**			
	**Normal BP**		**High BP** [c]		**Normal BP**		**High BP**		**Normal BP**		**High BP**		**Normal BP**		**High BP**	
	Difference (95% CI)	*p-value*	*Difference (95% CI)*	*p-value*	*Difference (95% CI)*	*p-value*	*Difference (95% CI)*	*p-value*	*Difference (95% CI)*	*p-value*	*Difference (95% CI)*	*p-value*	*Difference (95% CI)*	*p-value*	*Difference (95% CI)*	*p-value*
**MARS-5 sum score**																
crude [a]	-0.36 (-0.98 to 0.26)	0.250	0.27 (-0.27 to 0.81)	0.324	-0.24 (-0.96 to 0.49)	0.522	-0.11 (-0.80 to 0.58)	0.757	-0.33 (-1.05 to 0.39)	0.370	0.62 (-0.08 to 1.32)	0.081	-0.51 (-1.26 to 0.23)	0.178	0.35 (-0.39 to 1.08)	0.351
adjusted [b]	-0.32 (-0.94 to 0.32)	0.327	0.23 (-0.30 to 0.76)	0.397	-0.19 (-0.92 to 0.54)	0.611	-0.15 (-0.84 to 0.53)	0.658	-0.28 (-1.01 to 0.45)	0.447	0.59 (-0.10 to 0.89)	0.092	-0.46 (-1.22 to 0.30)	0.232	0.32 (-0.41 to 1.05)	0.394
	OR (95% CI)	*p-value*	*OR (95% CI)*	*p-value*	*OR (95% CI)*	*p-value*	*OR (95% CI)*	*p-value*	*OR (95% CI)*	*p-value*	*OR (95% CI)*	*p-value*	*OR (95% CI)*	*p-value*	*OR (95% CI)*	*p-value*
**MARS-5 (<25)**																
crude	1.15 (1.01 to 1.32)	0.039	0.99 (0.87 to 1.14)	0.939	1.05 (0.88 to 1.25)	0.612	1.09 (0.91 to 1.30)	0.359	1.12 (0.95 to 1.33)	0.188	0.93 (0.78 to 1.10)	0.392	1.33 (1.11 to 1.60)	0.003	0.97 (0.82 to 1.15)	0.722
adjusted	1.14 (0.99 to 1.30)	0.069	1.01 (0.88 to 1.15)	0.881	1.03 (0.86 to 1.23)	0.742	1.11 (0.93 to 1.32)	0.262	1.10 (0.94 to 1.30)	0.233	0.94 (0.80 to 1.11)	0.475	1.30 (1.08 to 1.58)	0.006	0.99 (0.82 to 1.18)	0.871

Abbreviation *CI: confidence interval, MARS-5: Medication Adherence Report Scale*

[a] *Corrected for baseline value of the particular outcome*

[b] *Additionally corrected for possible confounders age, gender and education level*

[c] *High blood pressure is defined as an elevated blood pressure exceeding 140 mm Hg in systolic phase and or 90 mm Hg in diastolic phase*

Table A5 Subgroup analyses including intervention participants with ≥3 barriers identified with the QBS during the first consultation (N=108)

	**Overall effect**		**T1**		**T2**		**T3**	
	Difference (95% CI)	*p-value*	*Difference (95% CI)*	*p-value*	*Difference (95% CI)*	*p-value*	*Difference (95% CI)*	*p-value*
**MARS-5 sum score**								
crude [a]	0.56 (-0.08 to 1.19)	0.087	0.19 (-0.57 to 0.96)	0.624	0.74 (-0.03 to 1.52)	0.060	0.84 (0.02 to 1.67)	0.044*
adjusted [b]	0.55 (-0.08 to 1.18)	0.086	0.17 (-0.58 to 0.93)	0.654	0.74 (-0.02 to 1.51)	0.058	0.84 (0.03 to 1.65)	0.042*
	OR (95% CI)	*p-value*	*OR (95% CI)*	*p-value*	*OR (95% CI)*	*p-value*	*OR (95% CI)*	*p-value*
**MARS-5 (<25)**								
crude	1.09 (0.98 to 1.21)	0.133	1.13 (1.00 to 1.28)	0.038*	1.00 (0.86 to 1.19)	0.921	1.12 (0.94 to 1.33)	0.198
adjusted	1.09 (0.98 to 1.22)	0.130	1.14 (1.00 to 1.30)	0.048*	1.01 (0.85 to 1.19)	0.903	1.12 (0.95 to 1.33)	0.180

Abbreviation *CI: confidence interval, MARS-5: Medication Adherence Report Scale, QBS: Quick Barrier Scan*

[a] *Corrected for baseline value of the particular outcome*

[b] *Additionally corrected for possible confounders age, gender and education level*

** Statistically significant*

Table A6 Subgroup analyses including participants with a MARS-5 score of ≤23 at baseline (N=102)

	**Overall effect**		**T1**		**T2**		**T3**	
	Difference (95% CI)	*p-value*	*Difference (95% CI)*	*p-value*	*Difference (95% CI)*	*p-value*	*Difference (95% CI)*	*p-value*
**MARS-5 sum score**								
crude [a]	0.35 (-0.39 to 1.09)	0.355	0.02 (-0.85 to 0.89)	0.966	0.52 (-0.36 to 1.40)	0.245	0.61 (-0.30 to 1.52)	0.187
adjusted [b]	0.43 (-0.30 to 1.15	0.252	0.09 (-0.76 to 0.95)	0.829	0.59 (-0.27 to 1.46)	0.179	0.68 (-0.21 to 1.58)	0.135
	OR (95% CI)	*p-value*	*OR (95% CI)*	*p-value*	*OR (95% CI)*	*p-value*	*OR (95% CI)*	*p-value*
**MARS-5 (≤23)**								
crude	0.98 (0.85 to 1.14)	0.816	1.10 (0.91 to 1.32)	0.314	0.91 (0.75 to 1.11)	0.364	0.93 (0.77 to 1.13)	0.483
adjusted	0.99 (0.85 to 1.14)	0.835	1.10 (0.91 to 1.33)	0.316	0.92 (0.76 to 1.11)	0.376	0.93 (0.77 to 1.13)	0.484

Abbreviation *CI: confidence interval, MARS-5: Medication Adherence Report Scale*

[a] *Corrected for baseline value of the particular outcome*

[b] *Additionally corrected for possible confounders age, gender and education level*
